# Health Literacy and Health Care System Confidence as Determinants of Attitudes to Vaccines in France: Representative Cross-Sectional Study

**DOI:** 10.2196/45837

**Published:** 2024-05-07

**Authors:** Georges Khoury, Jeremy K Ward, Julien Mancini, Amandine Gagneux-Brunon, Liem Binh Luong Nguyen

**Affiliations:** 1 CIC Cochin Pasteur Hôpital Cochin Assistance Publique—Hôpitaux de Paris Paris France; 2 Cermes3 CNRS, INSERM Université Paris Cité Paris France; 3 Cancer, Biomedicine & Society Group, APHM, Hop Timone, Public Health Department (BIOSTIC) INSERM, IRD, ISSPAM, SESSTIM Aix-Marseille University Marseille France; 4 Centre International de Recherche en Infectiologie, Team GIMAP Inserm, U1111, CNRS, UMR530 CHU de Saint-Etienne Saint-Etienne France

**Keywords:** vaccine hesitancy, health literacy, trust, attitude toward vaccines, public health, vaccination, COVID-19, adult, sociodemographic factor

## Abstract

**Background:**

Health literacy involves individuals’ knowledge, personal skills, and confidence to take action to evaluate and appraise health-related information and improve their health or that of their community.

**Objective:**

This study aimed to analyze the association between health literacy and attitude toward vaccines, adjusted with other factors.

**Methods:**

We used the SLAVACO Wave 3, a survey conducted in December 2021 among a sample of 2022 individuals, representative of the French adult population. We investigated factors associated with the attitude toward vaccines using respondents’ different sociodemographic data, health literacy levels, and the health care system confidence levels using a multinomial logistic regression analysis.

**Results:**

Among the participants, 440.4 (21.8%) were classified as “distrustful of vaccines in general,” 729.2 (36.1%) were “selectively hesitant,” and 852.4 (42.2%) were “nonhesitant.” In our model, the level of health literacy was not statistically different between the “distrustful of vaccines in general” and the “selectively hesitant” (*P*=.48), but it was associated with being a “nonhesitant” (adjusted odds ratio [aOR] 1.86, 95% CI 1.25-2.76). The confidence in the health care system was a strong predictor for a “nonhesitant” attitude toward vaccines (aOR 12.4, 95% CI 7.97-19.2). We found a positive correlation of 0.34 (*P*<.001) between health literacy and confidence in the health care system, but the interaction term between health literacy and health care system confidence was not significant in our model.

**Conclusions:**

Health literacy was associated with a “nonhesitant” attitude toward vaccines. The findings demonstrated that health literacy and confidence in the health care system are modestly correlated. Therefore, to tackle the subject of vaccine hesitancy, the main focus should be on increasing the population’s confidence and on increasing their health literacy levels or providing vaccine information addressing the needs of less literate citizens.

## Introduction

Vaccination is an essential component of primary health care, preventing over 20 potential fatal infectious diseases [1]. Vaccine hesitancy is defined as the refusal or hesitation in accepting vaccines when immunization services are available [2]. It has been designated by the World Health Organization [3] as one of the top 10 challenges in public health, underlining the need to research, understand, and tackle this global issue. It is dynamic and complex, affected by external factors such as time, location, and type of vaccine but also by individual factors such as complacency, convenience, and confidence [4,5]. Vaccine hesitancy is particularly strong in France, which is one of the most vaccine-hesitant countries in the world [6]. At the same time, France has reached a high level of vaccination coverage against COVID-19 [7]. During the COVID-19 vaccination campaign, many factors have been found to be strongly correlated with attitudes to vaccination in general and to vaccination against COVID-19, in particular, age, gender, complacency, political opinions, perception of one’s health status, trust in health authorities and professionals, and health literacy [8-10]. The ability of people to understand vaccine-related information as well as their understanding of medicine have focused much of the debates on the rise of vaccine hesitancy [11]. These observations resonate with the concept of “health literacy.” This term has emerged in the 1970s and refers to the motivation and skills used by individuals to access, understand, evaluate, and apply health information for their own health and that of their family and community; to make judgments and decisions about health care, prevention, and health promotion; and to maintain and promote quality of life throughout [12,13]. Health literacy includes abilities to evaluate and criticize health-related information; it involves a level of knowledge, personal skills, and confidence to take action to improve one’s personal health and the health of one’s community by changing one’s lifestyle and living conditions [14,15]. Low health literacy is directly associated with preventable undesirable health outcomes, including poorer general health, mortality, and inadequate decisions for preventive measures [16,17]. It has also been associated with a higher risk of being “hesitant” rather than “provaccination” [18].

Because health literacy bears heavily on people’s ability to access medical information and health care providers as well as their treatment of this information, it stands to reason that it constitutes a significant determinant of attitudes to vaccinations. However, very few papers have empirically investigated this relationship, which is far from straightforward [19]. Indeed, health literacy interplays with the components of the “3C” model, as described earlier.

Most studies assess functional health literacy mainly using tools like S-TOFHLA (Short Test of Functional Health Literacy in Adults), thereby focusing only on the ability to understand medical information [20,21]. This is restrictive compared with more recent definitions of health literacy, which include the ability to discuss and ultimately make decisions to promote one’s health, lacking the use of rigorously validated scales as we will in our research. This study aims to contribute comprehensively to the understanding of the relationship between health literacy and vaccine hesitancy. In this paper, we will not only investigate the role of health literacy but also the importance of trust in the health care system and their combined impact on vaccine hesitancy. By examining these factors simultaneously, we aim to offer a more holistic perspective on the phenomenon of vaccine hesitancy, ultimately contributing to more effective public health interventions and strategies.

## Methods

### Recruitment Procedure and Study Sample

Between December 2 and December 17, 2021, invitations to participate in the study were sent to 25,800 French adults, randomly selected from an digital panel of more than 700,000 respondents (provided by Bilendi [22]). The self-administered web-based questionnaires, lasting approximately 15 minutes, were completed during this period. Participants received remuneration in the form of points for completing these surveys. Ultimately, participants could exchange the accumulated points for gift cards, further acknowledging their invaluable contributions to the survey. We then used the quota sampling method to finally obtain a study sample of 2022 respondents matching the French mainland adult population in terms of age (18-24, 25-34, 35-49 50-64, 75+ years), sex (male and female), occupation (farmers, craftsmen, executives, intermediate professions, employees, workers, retirees, and other inactives), population size of the area of residence (<2000, 2000-20,000, 20,000-100,000, and >100,000 inhabitants), and region (Alsace, Aquitaine, Auvergne, Burgundy, Brittany, Center, Île-de-France, Languedoc, Nord-Pas-de-Calais, Normandy, Pays de la Loire, and Provence-Alpes-Côte d’Azur). A weighting procedure was applied to further match the sample to these characteristics when the quota was not perfectly met. To do so, we used the raking ratio method using the SAS Calmar macro developed in France by the National Institute of Statistics and Economic Studies.

### Data Collected and Outcome

After the participants’ consent was obtained, we collected information on respondents’ sociodemographic characteristics such as age, sex, educational attainment, and income, as well as their attitudes and practices on a number of issues including vaccination, politics, alternative medicine, and trust in various institutions. Our main outcome is the attitude of the French adult population toward vaccines, using a widely recognized typology for assessing attitudes toward vaccines in France [23,24]. We made a typology in 3 categories based on the answers to 5 questions with the same format, asking whether the responders were in favor of (1) vaccines in general, (2) the flu vaccine, (3) the hepatitis B virus (HBV) vaccine, (4) the human papillomavirus (HPV) vaccine, and (5) the measles vaccine. The categories were built as follows:

- Category 1 (nonhesitant): All respondents who answered favorably to all 5 questions (ie, individuals who are favorable for vaccines in general and 100% accepting all types of vaccines).
- Category 2 (provaccine but selectively hesitant): All respondents who answered “favorable” to the question on vaccines in general but who answered “unfavorable” or “I do not know” to at least 1 of the 4 questions on specific vaccines. These represent the people who are favorable to vaccines in general but have doubts or reservations about specific vaccines.
- Category 3 (distrustful of vaccines in general): All respondents who answered “unfavorable” to vaccines in general.

### Assessment of Health Literacy Level and Health Care System Confidence Level

We calculated 2 scores: a health literacy score (HLS_19_-Q12) and a confidence score. The health literacy score was based on a series of 12 questions with a 4-point Likert scale on the ability of each participant to understand, evaluate, and make health decisions in their everyday life using a 48-point score (Multimedia Appendix 1). A score between 4 and 48 was obtained summing the 12 responses, with a higher score indicating a higher health literacy level. We divided the population into quartiles for the analysis: “very high” for a score >36, “high” for a score between 32 and 36, “low” for a score between 26 and 31, and “very low” for a score <26. The health care system confidence score was based on a series of 5 questions about the confidence in science, government agencies that monitor health and environmental risks, government, physicians, and drug manufacturers using a 20-point score with a 4-point scale (Multimedia Appendix 2). A higher score indicates a higher confidence level. The confidence levels score was categorized as follows based on a quartile division: “confident” for a score ≥19, “somewhat confident” for a score between 17 and 19, “somewhat not confident” for a score between 13 and 16, and “not confident” for a score <13. To estimate the internal validity of our scores, we calculated the Cronbach coefficient, in which a result of >0.9 for the health literacy score and the confidence score was considered as a strong internal validity criterion.

### Statistical Analysis

Participants’ baseline characteristics were described for each level of vaccine hesitancy. We used chi-square test with Rao and Scott’s second-order correction in cross-tabulations, a univariate regression model to select statistically significant variables by a forward stepwise selection method (entry threshold *P*<.2), and a multinomial logistic regression to investigate factors associated with the attitude of the French adult population toward vaccines using respondents’ different background data, health literacy levels, and confidence levels. Our model was adjusted on gender, age, level of education, income, health care system confidence level, and health literacy level. Because of weighting, frequency counts including decimal points, odds ratios, and adjusted odds ratio (aOR) were reported with 95% CI. We also tested the first-order interaction between health literacy and confidence level to uncover hidden or conditional relationships in data that would not be apparent when looking at individual variables in isolation. A further sensitivity analysis was conducted based on our model excluding health care system confidence.

Pearson correlation tests were used to test the relationship between health literacy and health care system confidence to assess whether there is an association or linear relationship between them, which also provides information about the strength and direction of the association. This test will help us identify whether these 2 factors are positively, negatively, or not significantly associated with each other.

### Ethical Considerations

Informed consent to participate in this study (including analyses presented in this paper) was collected before the completion of the questionnaire. Researchers did not have access to identifying data on participants following standard practices for web-based surveys. Participants received points after completing the surveys. Ultimately, participants could exchange all the points they received as gift cards. The methodology of the study was reviewed and approved by the ethical committee of the INSERM (IRB00003888, #21-770).

## Results

### Characteristics of the Population

In our study population, 208.1 (10.3%) were young adults, 1276.3 (63.1%) were 25-64 years of age, and 537.4 (26.6%) were 64 years and older with a sex ratio of 91 male participants per 100 female participants. Regarding the level of education, 1322.5 (65.4%) achieved secondary education, and 743.4 (36.7%) earned less than €2000 per month (a currency exchange rate of €1=US $1.183 is applicable). Baseline characteristics of the study population, overall and by the level of vaccine hesitancy, are described in Table 1.

**Table 1:** Baseline demographics of study participants (SLAVACO Wave 3, 2021).

Variable		Total (N=2022), n (%)		Distrustful of vaccines in general (n=440.4), n (%)	Selectively hesitant (n=729.2), n (%)		Nonhesitant (n=852.4), n (%)		*P* value^a^
**Age** **(years)**									<.001
	18-24	208.1 (10.3)	56.2 (12.7)		56.84 (7.8)	95.2 (11.2)			
	25-34	295.9 (14.6)	87.6 (19.9)		98.1 (13.4)	110.3 (12.9)			
	35-49	487.5 (24.1)	132.8 (30.2)		156.7 (21.5)	198.0 (23.2)			
	50-64	492.9 (24.4)	94.6 (21.5)		198.9 (27.3)	199.5 (23.4)			
	65-74	290.3 (14.4)	41.6 (9.4)		117.9 (16.2)	130.8 (15.3)			
	75+	247.1 (12.2)	27.7 (6.3)		100.8 (13.8)	118.6 (13.9)			
**Sex**									<.001
	Male	962.5 (47.6)	193.5 (43.9)		319.0 (43.7)	450.1 (52.8)			
	Female	1059.5 (52.4)	246.9 (56.1)		410.2 (56.3)	402.3 (47.2)			
**Diploma**									<.001
	No degree	699.5 (34.6)	197.8 (44.9)		242.5 (33.3)	259.2 (30.4)			
	Baccalaureate	519.3 (25.7)	112.9 (25.6)		196.2 (26.9)	210.3 (24.7)			
	Bachelor	569.3 (28.2)	102.7 (23.3)		200.4 (27.5)	266.2 (31.2)			
	Postgraduate	233.9 (11.6)	27.0 (6.1)		90.1 (12.4)	116.8 (13.7)			
**Monthly salary (€)^b^**									<.001
	Less than €1000	189.2 (9.4)	46.4 (10.5)		65.8 (9)	77.0 (9)			
	€1000-€1500	250.6 (12.4)	75.6 (17.2)		75.9 (10.4)	99.1 (11.6)			
	€1500-€2000	303.3 (15)	75.6 (17.2)		104.2 (14.3)	123.5 (14.5)			
	€2000-€3000	461.7 (22.8)	88.7 (20.1)		197.4 (27.1)	175.7 (20.6)			
	€3000-€4000	330.9 (16.4)	52.3 (11.9)		134.6 (18.5)	144.1 (16.9)			
	More than €4000	249.3 (12.3)	30.6 (6.9)		74.1 (10.2)	144.7 (17)			
**Health literacy level**									<.001
	Very high (36+)	532.8 (26.3)	87.9 (20)		169.4 (23.2)	275.4 (32.3)			
	High (32-36)	457.1 (22.6)	77.4 (17.6)		166.6 (22.8)	213.1 (25)			
	Low (26-31)	519.4 (25.7)	108.5 (24.6)		197.7 (27.1)	213.2 (25)			
	Very low (<26)	512.7 (25.3)	166.6 (37.8)		195.5 (26.8)	150.6 (17.7)			
**Health care system confidence level**									<.001
	Confident (19+)	571.6 (28.3)	41.7 (9.5)		183.2 (25.1)	346.7 (40.7)			
	Somewhat confident (17-19)	322.3 (15.9)	42.8 (9.7)		120.0 (16.4)	159.5 (18.7)			
	Somewhat not confident (13-16)	684.6 (33.9)	170.6 (38.7)		259.9 (35.6)	254.1 (29.8)			
	Not confident (<13)	443.5 (21.9)	185.2 (42.1)		166.1 (22.8)	92.2 (10.8)			

^a^Chi-square test with Rao and Scott’s second-order correction.

^b^A currency exchange rate of €1=US $1.183 is applicable.

### Pattern of Vaccine Hesitancy in the Population

Among the 2022 participants, 852.4 (42.2%) were “nonhesitant,” 729.2 (36.1%) were “selectively hesitant” (among which, n=678.2, 93% were not favorable to 1 or 2 vaccines), and 440.4 (21.8%) were “distrustful of vaccines in general.” The “nonhesitant” group was older with a higher male or female sex ratio. Regarding health literacy score, the “nonhesitant” group had the highest proportion 275.4 (32.3%) with a “very high” score, whereas the “distrustful of vaccines in general” group had the highest proportion 166.6 (37.8%) with a “very low” score. Regarding confidence scores, the “nonhesitant” group had the highest proportion 346.7 (40.7%) of “confident” (>19/24), whereas the “distrustful of vaccines in general” group had the highest proportion 185.2 (42.1%) of “not confident” (<13/24). Of note, a large majority of “distrustful of vaccines in general” individuals 355.8 (80.8%) belong to either “somewhat not confident” or “not confident” categories.

### Factors Associated With Vaccine Hesitancy

We examined our multinomial logistic regression model factors associated with attitude toward vaccination taking the “distrustful of vaccines in general” group as a reference (Table 2). The sex ratio of male and female participants was not significantly different with the “selectively hesitant” group but was higher among the “nonhesitant” with an aOR of 1.43 (95% CI 1.09-1.88). Age was higher in the “selectively hesitant” population, and the aOR varied from 2.72 (95% CI 1.72-4.29) for the 50- to 64-year age group to 4.01 (95% CI 1.94-8.27) for the 75+-year age group. In the “nonhesitant” group, aOR passed from 1.93 (95% CI 1.16-3.20) for the 50- to 64-year age category to 3.28 (95% CI 1.57-6.86) for the 75+-year category. No association was found for the younger age group. The level of education was also associated with vaccine hesitancy: participants having a postgraduate degree yielded an aOR of 2.63 (95% CI 1.58-4.40) in the “selectively hesitant” group and an aOR of 2.21 (95% CI 1.33-3.68) in the “nonhesitant” group, taking the “distrustful of vaccines in general” as reference. Confidence was a strong predictor of the attitude toward vaccination in both categories, with a higher confidence score leading to a higher aOR. In the “selectively hesitant” category, aOR varied from 1.60 (95% CI 1.16-2.20) for “somewhat not confident” level to 4.13 (95% CI 2.64-6.46) for the “confident” level. In the “nonhesitant” group, aOR varied from 2.77 (95% CI 1.91-4.01) for the “somewhat not confident” level to 12.4 (95% CI 7.97-19.2) for the “confident” level. While the health literacy levels did not appear statistically significant in the “selectively hesitant” group, a statistical difference was observed in the “nonhesitant” group compared to the “distrustful of vaccines in general” group: aOR increased from 1.50 (95% CI 1.04-2.16) for the “low” level of health literacy to 1.69 (95% CI 1.14-2.50) for the “high” level of health literacy and eventually 1.86 (95% CI 1.25-2.76) for the “very high” level of health literacy.

**Table 2:** Multinomial logistic regression results: exploring determinants of vaccine attitudes and hesitancy in France (SLAVACO Wave 3, 2021; N=2022).

Characteristic		Selectively hesitant				Nonhesitant		
		aOR^a^ (95% CI)		*P* value	aOR (95% CI)			*P* value
Intercept		0.38 (0.20-0.74)		*.005* ^b^	0.19 (0.09-0.38)			*<.001*
**Sex**								
	Female	Reference	Reference		Reference		Reference	
	Male	0.99 (0.73-1.36)	>.88		1.43 (1.09-1.88)		*.01*	
**Age (years)**								
	18-24	Reference	Reference		Reference		Reference	
	25-34	1.20 (0.73-1.97)	.50		0.98 (0.56-1.71)		>.90	
	35-49	1.40 (0.85-2.30)	.21		1.26 (0.76-2.12)		.39	
	50-64	2.72 (1.72-4.29)	*<.001*		1.93 (1.16-3.20)		*.01*	
	65-74	3.05 (1.73-5.38)	*<.001*		2.25 (1.23-4.14)		*.01*	
	75+	4.01 (1.94-8.27)	*<.001*		3.28 (1.57-6.86)		*.002*	
**Degree**								
	No degree	Reference	Reference		Reference		Reference	
	Baccalaureate	1.59 (1.10-2.31)	*.01*		1.56 (1.08-2.24)		*.02*	
	Undergraduate	1.67 (1.18-2.37)	*.004*		1.82 (1.27-2.61)		*.001*	
	Postgraduate	2.63 (1.58-4.40)	*<.001*		2.21 (1.33-3.68)		*.003*	
**Monthly salary (€)^c^**								
	Less than €1000	Reference	Reference		Reference		Reference	
	€1000-€1500	0.62 (0.34-1.13)	.12		0.71 (0.37-1.35)		.28	
	€1500-€2000	0.76 (0.45-1.31)	.29		0.82 (0.47-1.43)		.54	
	€2000-€3000	1.04 (0.61-1.75)	.87		0.76 (0.45-1.30)		.26	
	€3000-€4000	1.09 (0.61-1.87)	.83		0.91 (0.53-1.57)		.71	
	More than €4000	0.83 (0.43-1.61)	.57		1.22 (0.65-2.32)		.52	
**Health care system confidence level**								
	Not confident (<13)	Reference	Reference		Reference		Reference	
	Somewhat not confident (13-16)	1.60 (1.16-2.20)	*.005*		2.77 (1.91-4.01)		*<.001*	
	Somewhat confident (17-19)	2.88 (1.77-4.71)	*<.001*		6.51 (4.13-10.3)		*<.001*	
	Confident (19+)	4.13 (2.64-6.46)	*<.001*		12.4 (7.97-19.2)		*<.001*	
**Health literacy level**								
	Very low (<26)	Reference	Reference		Reference		Reference	
	Low (26-31)	1.22 (0.84-1.77)	.27		1.50 (1.04-2.16)		*.03*	
	High (32-36)	1.27 (0.85-1.90)	.19		1.69 (1.14-2.50)		*.009*	
	Very high (36+)	1.13 (0.77-1.67)	.48		1.86 (1.25-2.76)		*.003*	

^a^aOR: adjusted odds ratio.

^b^Values in italics format are statistically significant with a *P*<.05.

^c^A currency exchange rate of €1=US $1.183 is applicable.

The robustness of these results was tested by conducting a multivariable regression model for each type of vaccine included in the questionnaire (vaccine in general, flu vaccine, HPV vaccine, HBV vaccine, and measles vaccine) with the same covariables and found that for each vaccine, health care system confidence was highly associated with a positive attitude toward vaccine (*P*<.001), but health literacy was no longer associated with a positive attitude toward each vaccine (*P*>.05) with no interaction term between these 2 variables (*P*>.10). The results of the Pearson correlation coefficient showed a modest positive correlation of 0.34 (*P*<.001) between health literacy and the health care system confidence.

To explore these findings, our multivariable regression model for each of the vaccination types listed in the questionnaire (vaccine in general, flu vaccine, HPV vaccine, HBV vaccine, and measles vaccine) showed that in all of the regression models, vaccine acceptance for each of the vaccines mentioned was strongly associated with the confidence level but not with health literacy with no interaction between health literacy and confidence (*P* value of interaction >.1). We found a modest correlation (Pearson correlation coefficient=0.34) between health literacy and confidence. Another multinomial logistic regression was performed by removing the health care system confidence indicator from our model (Table 3). In this model, the effect of health literacy on vaccine hesitancy appeared to be significant (*P*<.001) with higher aOR.

**Table 3:** Sensitivity analysis on multinomial logistic regression model: exploring determinants of vaccine attitudes and hesitancy in France (SLAVACO Wave 3, 2021; N=2022) with the exclusion of the health care system confidence variable.

Characteristic		Selectively hesitant				Nonhesitant		
		aOR^a^ (95% CI)		*P* value	aOR (95% CI)			*P* value
Intercept		0.63 (0.34-1.16)		.13	0.54 (0.31-0.95)			*.03* ^b^
**Sex**								
	Female	Reference	Reference		Reference		Reference	
	Male	0.98 (0.77-1.26)	.87		1.43 (1.11-1.83)		*.006*	
**Age (years)**								
	18-24	Reference	Reference		Reference		Reference	
	25-34	0.98 (0.60-1.60)	>.90		0.63 (0.42-1.08)		.10	
	35-49	1.18 (0.75-1.84)	.54		0.94 (0.60-1.49)		.78	
	50-64	2.33 (1.48-3.66)	*<.001*		1.49 (0.99-2.23)		.05	
	65-74	2.87 (1.55-5.29)	*.001*		2.03 (1.15-3.61)		*.02*	
	75+	3.60 (2.01-6.48)	*<.001*		2.76 (1.54-4.95)		*<.001*	
**Degree**								
	No degree	Reference	Reference		Reference		Reference	
	Baccalaureate	1.53 (1.04-2.25)	*.03*		1.44 (1.02-2.05)		*.04*	
	Undergraduate	1.73 (1.21-2.46)	*.003*		1.92 (1.34-2.75)		*<.001*	
	Postgraduate	2.83 (1.58-5.08)	*<.001*		2.54 (1.45-4.42)		*.001*	
**Monthly salary (€)^c^**								
	Less than €1000	Reference	Reference		Reference		Reference	
	€1000-€1500	0.61 (0.34-1.08)	.08		0.70 (0.38-1.27)		.20	
	€1500-€2000	0.75 (0.45-1.26)	.32		0.82 (0.47-1.44)		.51	
	€2000-€3000	1.09 (0.68-1.76)	.68		0.85 (0.51-1.41)		.54	
	€3000-€4000	1.11 (0.65-1.91)	.70		0.96 (0.58-1.62)		.88	
	More than €4000	0.87 (0.43-1.78)	.66		1.36 (0.90-2.66)		.41	
**Health literacy level**								
	Very low (<26)	Reference	Reference		Reference		Reference	
	Low (26-31)	1.42 (1.01-1.99)	*.04*		1.98 (1.40-2.81)		*<.001*	
	High (32-36)	1.74 (1.16-2.60)	*.008*		2.92 (1.97-4.35)		*<.001*	
	Very high (36+)	1.57 (1.10-2.24)	*.01*		3.33 (2.35-4.73)		*<.001*	

^a^aOR: adjusted odds ratio.

^b^Values in italics format are statistically significant with a *P*<.05.

^c^A currency exchange rate of €1=US $1.183 is applicable.

## Discussion

### Principal Findings

This study confirms a relationship between vaccine hesitancy and health literacy in this study sample, representative of the French population, in which only 852.4 (42.2%) are “nonhesitant.” In a regression model looking at factors associated with vaccine hesitancy, we found that higher age, male sex, health care system confidence, health literacy, and educational attainment were independently associated with less vaccine hesitancy. Health care system confidence was notably a strong predictor: a person with the highest level of confidence is 12 times less likely to be “distrustful of vaccines in general” and 4 times less likely to be “selectively hesitant.”

The level of vaccine hesitancy in this study sample is consistent with other studies conducted in France [23,24]. Our regression model confirms the “confidence, complacency, constrain” (so-called “3C”) model developed by the Strategic Advisory Group of Experts on Immunization Working Group [2]. However, confidence is multileveled: it includes confidence in health care professionals but also in the health care system, and the national state. This study specifically addressed confidence in the health care system. While primary care providers are often among the most trusted, confidence in the state and policy makers can be much more difficult to build or regain. In this study, we found a widespread mistrust of government and the pharmaceutical industry that logically translates into mistrust of vaccines (n=355.8, 80% of the “distrustful of vaccines in general” participants belonged to the nonconfident categories).

We also looked at the role of health literacy in vaccine confidence. A recent systematic review that looked at the association of health literacy with intention to vaccinate and vaccination status found high heterogeneity in the assessment of health literacy, inconsistent results, or a weak association when analyzing 21 papers [19]. Studies have hypothesized a mediating role of health literacy on the relationship between health care system distrust and vaccine hesitancy [25], while in other context, researchers studied, for example, the potential mediating effect of trust in one’s physician on the association of health literacy and medication adherence [26]. This could reflect the fact that health literacy is often associated with other properties that are also likely to affect attitudes to vaccines such as mistrust of actors in vaccination policies, but it could also reflect the paradoxical effect that health literacy is likely to have on attitudes to vaccines. Indeed, health literacy can both help to navigate the health care system and gain access to medical professionals who provide evidence-based information, but it can also favor “doing your own research” and coming across vaccine-critical information. This potentially paradoxical effect of the ability and propensity to look for information regarding health has been well documented by work on the relationship between healthism—the tendency for individuals to exercise control over their own choices in order to maximize their life expectancy—and vaccine hesitancy [27]. Our results contribute to these reflections. We found that a higher health literacy level is associated with being “nonhesitant,” whereas it was not statistically significant in selective hesitancy when controlling for confidence in health care actors.

The relationship between confidence in the health care system and health literacy was also examined. We found that these 2 factors were independent in our model (no interactions found) but modestly correlated. The correlation between the 2 variables seems to be less strong in France than in other countries. For instance, a cross-sectional survey conducted in Taiwan to measure the association between health literacy and trust in physicians and health care systems with a population of 2199 adults showed that respondents’ level of confidence in medical professionals and the health care system was higher among those with better health literacy [28]. After adjusting for respondents’ sociodemographic factors, health literacy remained significantly and positively associated with confidence. Our results therefore suggest that literacy in itself might be less important in France compared to trust. This could reflect the fact that distrust of public actors is particularly strong in France. Comparative work could shed light on national differences in the relationship between trust in health care actors and health literacy. The relationship between the 2 is likely to be affected—among other factors—by the organization of health care and how social inequalities are reflected in health. The absence of interaction effect between health literacy and trust in health care actors is also interesting. We could expect that a higher level of literacy combined with a higher distrust of health care actors would favor the tendency to find and appreciate vaccine-critical sources. It is possible that these tendencies are counterbalanced by a greater propensity among the more literate to identify the public signals denouncing the antivaccine rhetoric as antiscientific, which tend to be pervasive in the mainstream media, especially in France [29,30]. One possible avenue of research in future work could be to explore the relationship between health literacy and information-seeking practices on social media. Inspiration could come from research on how political attitudes interact with social media use and with educational attainment in the inception of vaccine attitudes (for reviews of the literature, see [31,32]).

We found that the male sex was associated with the “nonhesitant” group (aOR 1.43, 95% CI 1.09-1.88). These findings confirm that male sex had more often positive attitudes toward vaccines, whereas women are more likely to be hesitant. These findings are in line with previous studies on vaccine hesitancy [33-35].

Level of education and educational attainment were found to be an independent factor on vaccine acceptance after adjusting for health literacy and health care system confidence.

Finally, in our model, the level of income did not appear to be statistically significant with vaccine acceptability. Despite not achieving statistical significance in the multinomial regression model, it is worth noting that the estimated aORs for vaccine hesitancy showed a trend to enhance vaccine acceptance with a salary in the range of €2000-€4000 per month for the “selectively hesitant” group and for a salary of more than €4000 per month for the “nonhesitant” group. These results confirm prior study results, which indicates that families in the lowest income categories in Brazil, which account for one-third of the population, are reported to be the most vaccine hesitant [36]. Given that those in lower income brackets (income <€1500 per month) make up about 15% of the French population in 2018 [37] and showed the lowest vaccination acceptance rates (23.05%) and the greatest vaccine hesitancy rates (33.04%) [38].

### Limitations

This study also has several limitations: health literacy is complex. Our questionnaires using close-ended questions may not capture the whole dimensions of this concept. For example, our questionnaire could not separate health literacy from scientific literacy. The same limitation applies for confidence. These limits could only be resolved using qualitative methods that could examine how health literacy and confidence translate in reality with vaccine providers. The second limitation of this study was the recruitment procedure, which uses quota sampling and can lead to more biases than random sampling. However, the frequency of vaccine hesitancy appeared to be consistent with the frequency previously reported in the literature.

### Strengths

Finally, this study has several strengths: the study population was representative of the French population, which has one of the highest vaccine hesitancy rates; we used a validated tool to assess vaccine hesitancy; and the large sample size of this study allows to examine various factors that have been separately associated with vaccine hesitancy in previous studies as well as different vaccines.

### Conclusions

Interventions aiming to reduce vaccine hesitancy should prioritize confidence-building measures, which are difficult. Improving health literacy may also help, and public health interventions demonstrated their ability to increase health literacy. We believe that there is a possibility that improving health literacy can also lead to enhanced confidence in the health care system in the long run. Public health campaigns and educational programs should enhance health literacy, while health care providers engage in open, empathetic vaccine discussions. Community leaders and organizations play a role in promoting vaccination. Further research on the relationship between confidence and health literacy is essential. Transparency, timely updates, and public-private partnerships are vital. Tailored interventions should address gender disparities and involve continuous monitoring. Fostering understanding, trust, and tailored strategies can enhance vaccine acceptance and coverage in France.

## Appendix

Multimedia Appendix 1 Health literacy questionnaire (HLS19-Q12).

Multimedia Appendix 2 Health care system confidence questionnaire.

## Abbreviations

aOR: adjusted odds ratio
HBV: hepatitis B virus
HPV: human papillomavirus
S-TOFHLA: Short Test of Functional Health Literacy in Adults

## Data Availability

The data sets analyzed during this study are available in the Harvard Dataverse repository [39].
