# Supplementary material for: Health Literacy and Health Care System Confidence as Determinants of Attitudes to Vaccines in France: Representative Cross-Sectional Study
Source: JMIR Public Health Surveill. 2024 May 7;10:e45837. doi: 10.2196/45837 (PMC11109853; doi:10.2196/45837)
Supplement: Multimedia Appendix 1 [file publichealth_v10i1e45837_app1.docx]

The HLS_19_ instrument used in this research was developed within “HLS_19_ – the International Health Literacy Population Survey 2019-2021” of M-POHL. An application including a contractual agreement with the conditions for using the HLS_19_ instruments has to be provided. A template for the application and agreement can be found at https://m-pohl.net/tools.

On a scale from very easy to very difficult, how easy would you say it is: (very easy/easy/difficult/very difficult)

…to find out where to get professional help when you are ill? [Instructions: such as doctor, nurse, pharmacist, psychologist]"

…to understand information about what to do in a medical emergency?

…to judge the advantages and disadvantages of different treatment options?

...to act on advice from your doctor or pharmacist?

…to find information on how to handle mental health problems? [Instruction: stress, depression or anxiety]"

…to understand information about recommended health screenings or examinations? [Instructions: e.g. colorectal cancer screening, blood sugar test, blood pressure]"

…to judge if information on unhealthy habits, such as smoking, low physical activity or drinking too much alcohol, are reliable?

…to decide how you can protect yourself from illness using information from the mass media? [Instructions: e.g. Newspapers, TV or Internet]"

…to find information on healthy life styles such as physical exercise, healthy food or nutrition?

…to understand advice concerning your health from family or friends?

...to judge how your housing conditions may affect your health and well-being?

…to make decisions to improve your health and well-being?
