# Supplementary material for: Health Literacy and Health Care System Confidence as Determinants of Attitudes to Vaccines in France: Representative Cross-Sectional Study
Source: JMIR Public Health Surveill. 2024 May 7;10:e45837. doi: 10.2196/45837 (PMC11109853; doi:10.2196/45837)
Supplement: Multimedia Appendix 2 [file publichealth_v10i1e45837_app2.docx]

Do you trust or not trust...?

(Very confident / Somewhat confident / Not very confident/ Not at all confident / Do not know or do not wish to answer this question)

● Science

● Government agencies that monitor health and environmental risks

● The government

● Physicians

● The drug manufacturers
